# Correlation of ACE2 with RAS components after Losartan treatment in light of COVID-19

**DOI:** 10.1038/s41598-021-03921-5

**Published:** 2021-12-22

**Authors:** Javeria Zaheer, Hyeongi Kim, Jin Su Kim

**Affiliations:** 1 Division of RI Application, Korea Institute of Radiological and Medical Sciences, 75 Nowon-Gil, Gongneung-Dong, Nowon-Gu, Seoul, 01812 Korea; 2 Radiological and Medico-Oncological Sciences, University of Science and Technology (UST), Seoul, 01812 Korea

**Keywords:** Health care, Medical research

## Abstract

Angiotensin-converting enzyme 2 (ACE2) is an important factor in coronavirus disease (COVID-19) interactions. Losartan (LOS) belongs to the angiotensin receptor blocker (ARB) family. Additionally, the protective role of ACE2 restored by LOS has been suggested and clinically examined in the treatment of COVID-19 patients. Furthermore, clinical trials with LOS have been conducted. However, the mechanism through which LOS enhances ACE2 expression remains unclear. In addition, the response of ACE2 to LOS differs among patients. Our LOS-treated patient data revealed a correlated mechanism of ACE2 with components of the renin-angiotensinogen system. We observed a significant positive regulation of MAS1 and ACE2 expression. In the context of LOS treatment of COVID-19, ACE2 expression could depend on LOS regulated MAS1. Thus, MAS1 expression could predict the COVID-19 treatment response of LOS.

## Introduction

ACE2 is an important factor in coronavirus disease (COVID-19) interactions, because the virus uses the ACE2 receptor to gain entry into the cell^1,2^. Losartan (LOS) belongs to the angiotensin receptor blocker (ARB) family^3^. The beneficial roles of ARBs or ACE blockers against COVID-19 were reported^4,5^. The LOS was suggested and clinically assessed in the treatment of COVID-19 patients (clinical trials NCT04335123, NCT04311177, and NCT04312009)^1^. However, the response of ACE2 to LOS differed among studies. Carols et al. showed an increase in ACE2 levels with LOS^6^. It has been reported that LOS treatment does not affect changes in ACE2^7^. The studies represent controversial response of LOS in context of ACE2.

In this study, we identified the correlation analysis of RAS components after LOS treatment and analyzed the possible reasons for the individual differences in ACE2 expression in patients before and after LOS treatment. To analyze the correlation of ACE2 upregulation, public data were accessed using the NCBI Gene Expression Omnibus dataset (GEO database set) with the query dataset for GSE37824. The data were analyzed with GEO2R by defining groups for LOS baseline and LOS 6 week’s treatment in the LOS arm. The gene expression of samples was first assessed for the groups, normalized with GAPDH, and further analyzed for log2 (fold-change) in individual ACE2 expression to identify individual patient’s differences at baseline and 6 weeks of LOS-treated patients in the LOS arm.

## Results

### Response of ACE2 to LOS differed among patients

**Figure 1:**
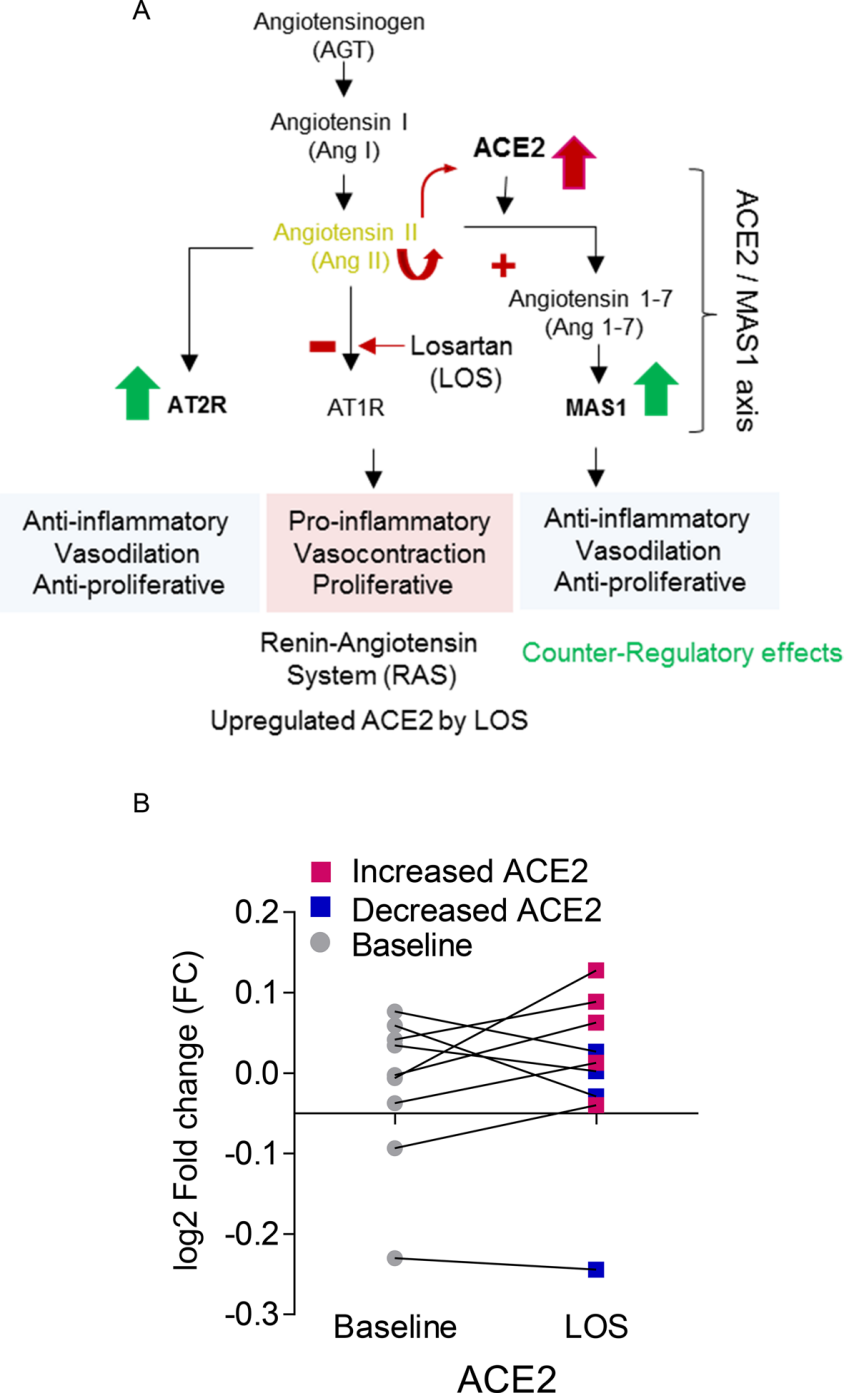
Response of ACE2 to LOS differed among patients. (**A**) Schematic diagram illustrates the upregulation of ACE2 after LOS treatment. (**B**) ACE2 log2 fold change expression levels of individual patient at baseline and 6 weeks after LOS treatment.

Figure 1A shows schematic diagram of correlation of losartan (LOS)-mediated upregulation of ACE2. All patients received LOS 50 mg. As shown in Fig. 1B, 66% of patients showed increased ACE2, where 34% shows decreased ACE2 log2 fold change from baseline.

### Response of RAS components to LOS among patients

**Figure 2:**
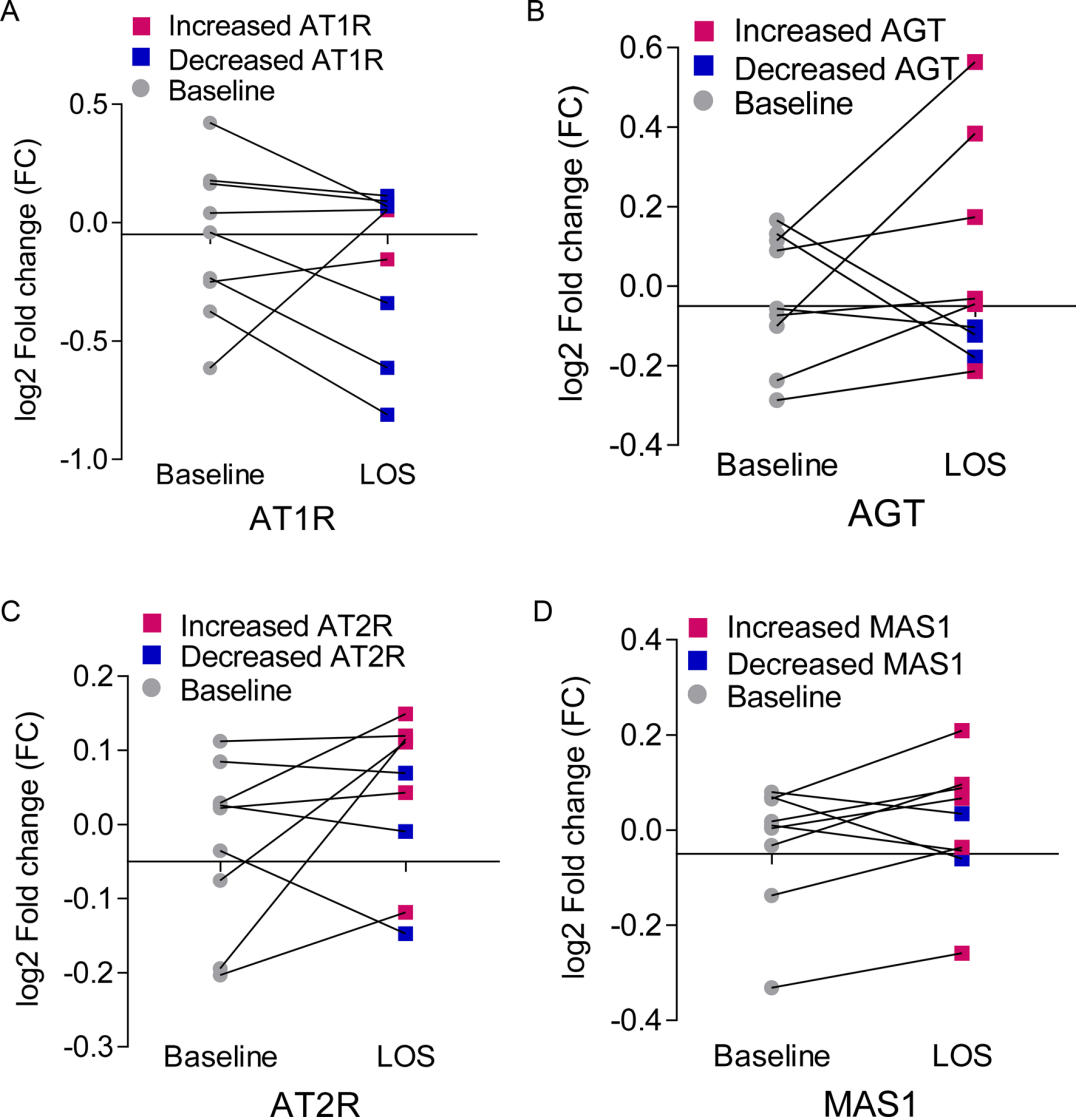
Response of RAS components to LOS among patients. (**A**) Increase and decrease response of AT1R of individual patients at baseline and at 6 weeks of LOS treatment. (**B**) Increase and decrease response of AGT of individual patients at baseline and at 6 weeks of LOS treatment. (**C**) Increase and decrease response of AT2R of individual patients at baseline and at 6 weeks of LOS treatment. (**D**) Increase and decrease response of MAS1 of individual patients at baseline and at 6 weeks of LOS treatment.

LOS target receptor is AT1R^3^. We analyzed renin angiotensin system (RAS) components AT1R, AGT, AT2R and MAS1 to evaluate the LOS mediated response. We observed 77% of patients showed decreased AT1R, and 23% showed increase AT1R log2 fold change from baseline Fig. 2A. As for other RAS components we observed 66.6% showed increased angiotensinogen (AGT) and 34% shows decreased AGT log2 fold change from baseline as shown in Fig. 2B. Similarly, angiotensin type 2 receptor (AT2R) shows 66% increased and 34% decreased log2 fold change from baseline (Fig. 2C). Additionally, 77% showed increased levels of MAS1 proto-oncogene (*MAS1*) at 6 weeks of LOS treatment, compared to baseline (Fig. 2D).

### Negative Pearson correlation of RAS component to LOS treatment

**Figure 3:**
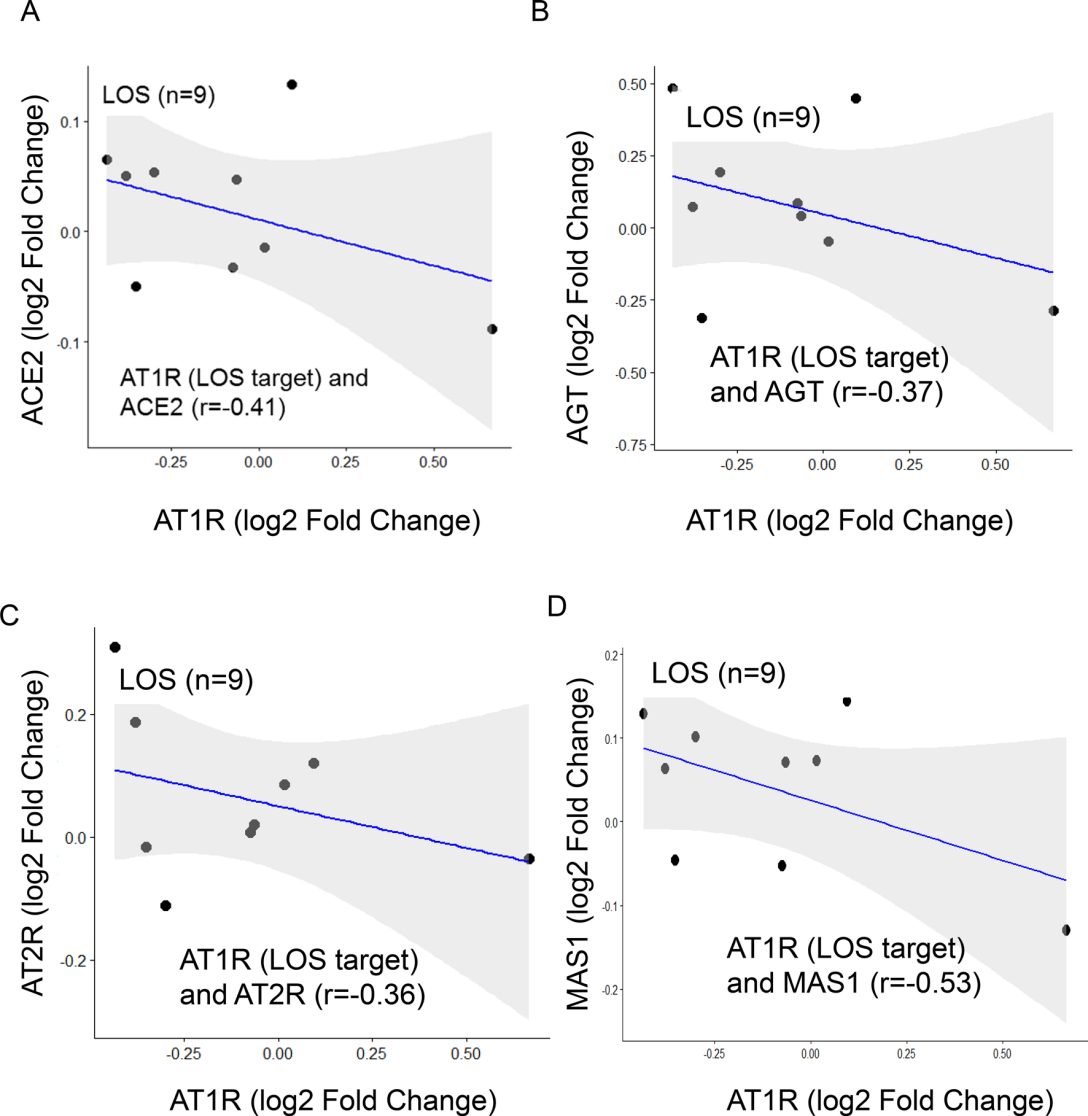
Negative Pearson correlation of RAS component to LOS treatment. (**A**) Negative correlation of AT1R with ACE2 (r = − 0.41). (**B**) Negative correlation of AT1R with AGT (r = − 0.37). (**C**) Negative correlation of AT1R with AT2R (r = − 0.36). (**D**) Negative correlation of AT1R with MAS1 (r = − 0.53). Calculation of correlation coefficient and testing the significance of the correlation coefficient were performed using R (* denotes P < 0.005).

Moreover, we performed correlation analysis of LOS target receptor AT1R with RAS component. A moderate correlation was observed between levels of AT1R and ACE2, (r = − 0.41), AT1R and AGT (r = − 0.37), AT1R and AT2R (r = − 0.36) and AT1R and MAS1 (r = − 0.53) as shown in Fig. 3A–D.

### Positive Pearson correlation of RAS component to LOS treatment

**Figure 4:**
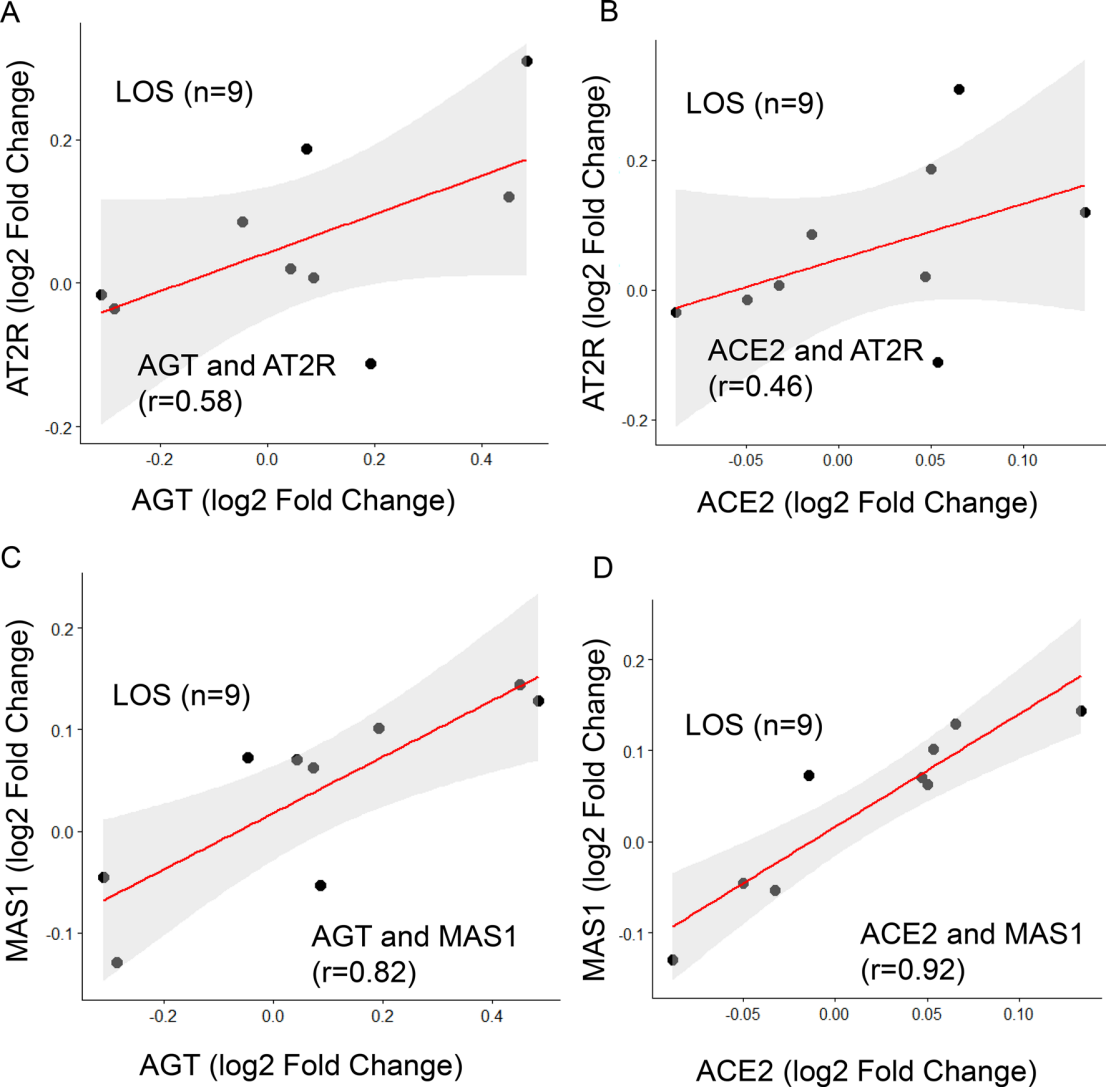
Positive Pearson correlation of RAS component to LOS treatment. (**A**) Positive correlation of AGT with AT2R (r = 0.58). (**B**) Positive correlation of ACE2 with AT2R (r = 0.46). (**C**) Positive correlation of AGT with MAS1 (r = 0.82, ***P < 0.001). (**D**) Positive correlation of ACE2 with MAS1 (r = 0.92, ***P < 0.001). Calculation of correlation coefficient and testing the significance of the correlation coefficient were performed using R (* denotes P < 0.005).

A moderate correlation was observed for the gene pairs *AGT* and *AT2R* (r = 0.58) represented in Fig. 4A, between *ACE2* and *AT2R* (r = 0.46) shown in Fig. 4B, and strong correlation between *AGT* and *MAS1* (r = 0.82, *P* = 0.007) as shown in Fig. 4C and between ACE2 and MAS1 (r = 0.92, P = 0) as shown in Fig. 4D.

## Discussion

In this study, we identified the LOS regulated MAS1 expression. We observed significant positive correlations between AGT and MAS1 expression (Fig. 4C) and between MAS1 and ACE2 expression (Fig. 4D). MAS1 is involved in the final step of ACE2 upregulation^8,9^. This positive correlation of AGT and ACE2 with MAS1 could confirm the increased demand for ACE2 in LOS-treated patients.

The increased AT1R levels after LOS treatment could be due to either a negative trend or non-responsiveness to LOS treatment at the given dose. The physiological role of AT2R and MAS1 is opposite to that of AT1R (Fig. 1A), which induces a counter-regulatory effect^10^. MAS1 expression was moderately negatively correlated with AT1R expression (Fig. 3D), suggesting that MAS1 is activated after LOS treatment. We observed a negative moderate correlation between AT1R and AGT expression (Fig. 3B), which could suggest LOS-mediated inhibition of AT1R could increase synthesis of Ang II (or AGT), which competes with LOS for AT1R. We observed a positive trend between AT2R and ACE2 levels (Fig. 4B), suggesting the activation of AT2R after LOS treatment. However, the trend was not significant. Therefore, more clinical records of COVID-19-infected patients with larger number of patients undergoing LOS therapy are needed to further elucidate the counterbalance effect of AT1R and AT2R.

**Figure 5:**
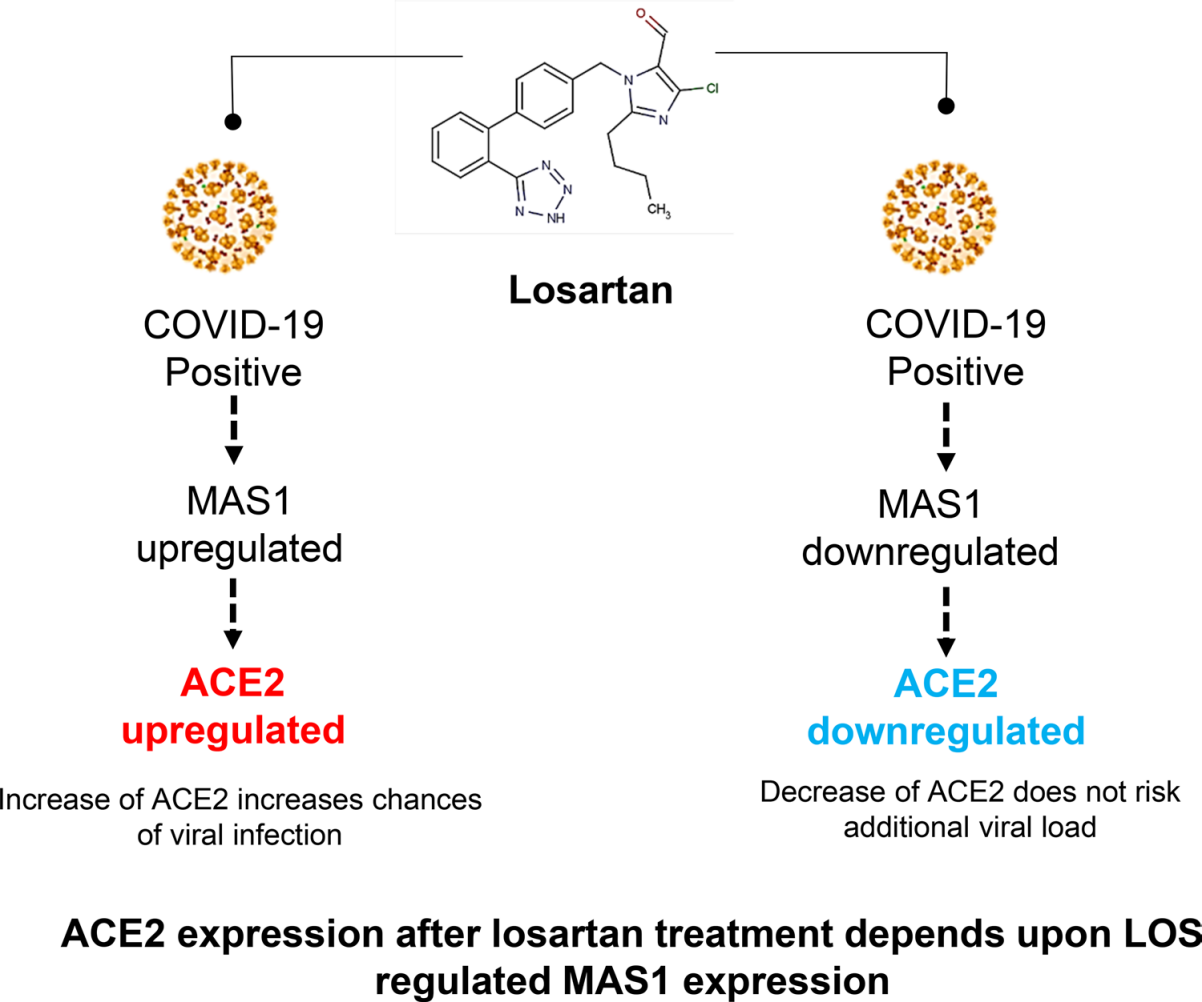
Schematic diagram shows controversy of losartan treatment for Covid-19. Determining the MAS1 expression could predict the response to SARS-CoV-2 viral load for patients who are already on LOS medication for pre-existing condition.

Recent studies have shown that COVID-19 patients use more ARBs and ACEIs due to cardiovascular complication. Till now there is no evidence that ARBs heightened the risk of COVID-19^11–15^. In this study, we revealed the possible reason for the controversial response of LOS in the context of ACE2 (Fig. 5). LOS has shown controversial response at clinical study^6,7^. SARS-CoV-2 uses ACE2 to gain cell entry. Increase of ACE2 with LOS could risk the viral load however the decrease of ACE2 could compromise the protective function of ACE2 with no additional viral load. Here we projected ACE2 expression depends upon the LOS regulated expression of MAS1 by utilizing human data.

In sum, concerning the treatment of COVID-19 with LOS, ACE2 expression is associated with MAS1. MAS1 expression could predict the COVID-19 treatment response of LOS. SARS-CoV-2 binds to ACE2, enabling cell entry. Thus determining MAS1 expression could predict the response to SARS-CoV-2 viral load for patients who are already on LOS medication for a pre-existing condition.

## Methods

### Public data usage and accordance statement

Publicly available NCBI Gene Expression Omnibus dataset GSE37824 has been used in study. NCBI itself places no restrictions on the use or distribution of the data contained therein. Nor do it accept data when the submitter has requested restrictions on reuse or redistribution. The human study used in our study was sourced by Medtronic Endovascular with clinical trial NCT no: NCT00720577.

## Public data

### NCBI gene dataset

To analyze the mechanism of ACE2 upregulation, public data were accessed using the NCBI Gene Expression Omnibus dataset (GEO database set) with the query dataset for GSE37824 (https://www.ncbi.nlm.nih.gov/geo/geo2r/?acc=GSE37824). Patients with peripheral artery disease were enrolled and randomized into 6 weeks of drug treatment vs baseline in LOS treated arm for paired analysis (n = 9). Patients underwent catheter excision of atherosclerotic tissue from one extremity at baseline and the contralateral extremity after treatment. 50 mg LOS once a day was given which reduced diastolic blood pressure by 4 mm Hg (p = 0.099). Sample were analyzed for expression profiling by array using Rosetta/Merck human 44 k 1.1 microarray platform. The gene expression of samples was first assessed for the groups, normalized with GAPDH, and further analyzed for log2 fold change in individual ACE2 expression through paired analysis at baseline and 6 weeks of treatment. The data were analyzed with GEO2R by defining groups for LOS baseline and LOS 6 week’s treatment in the LOS arm. The gene expression of samples was first assessed for the groups, normalized with GAPDH, and further analyzed for log2 fold change in individual ACE2 expression to identify individual patient’s differences at baseline and 6 weeks of LOS-treated patients in the LOS arm for gene ACE2, AT1R, AT2R, AGT and MAS1 for LOS treated samples.

Correlation analysis was performed using the R software. The correlation between LOS and AT1R, AGT, AT2R, or MAS1 was analyzed. The correlation between AT1R and ACE2, AGT, or AT2R was analyzed. In addition, the correlation between AGT and AT2R, between ACE2 and AT2R, between AGT and MAS1 was analyzed.

### Statistical analysis

Calculation of correlation coefficient were performed using R.

### Declaration

Because we used NCBI Gene Expression Omnibus dataset to reveal the correlation between ACE2, ACE2, AT1R, AT2R, AGT and MAS1 for LOS treated samples, it is not required to identify the institutional and/or licensing committee approving the experiments, including any relevant details. In addition, it is also not required to declare documentation related to informed consent, ethics approval and donor organ/tissue source, including approved translations.

## Data Availability

All data are included in the manuscript.

## Abbreviations

ACE2: Angiotensin-converting enzyme 2
Ang II: Angiotensin II
AGT: Angiotensinogen
MAS1: Mas proto-oncogene gene
AT1R: Angiotensin receptor 1
AT2R: Angiotensin receptor 2
LOS: Losartan
COVID19: Coronavirus disease 2019
